# Adverse Physical Consequences of Perinatal E-cigarette Use by Pregnant Mothers on Their Offspring: A Scoping Review

**DOI:** 10.7759/cureus.102109

**Published:** 2026-01-22

**Authors:** Yatin Srinivash Ramesh Babu, Ambika M Kapil, Andrew R Gunthner, Rohan V Rajan, Angeline Triyono, Andrew Schafer, Austin M Chen, Kyle M Maisel, Hamid Gogerdchian, Jackson Copper, Joshua M Costin

**Affiliations:** 1 Department of Medicine, Nova Southeastern University Dr. Kiran C. Patel College of Osteopathic Medicine, Fort Lauderdale, USA; 2 Department of Medical Education, Nova Southeastern University Dr. Kiran C. Patel College of Allopathic Medicine, Fort Lauderdale, USA

**Keywords:** congenital abnormalities, e-cigarette use, maternal vaping, neonatal health outcomes, nicotine, perinatal health, pregnancy, vaping

## Abstract

E-cigarette use among pregnant women has increased as a smoking cessation method, raising public health concerns about fetal chemical exposure, yet most research relies on animal models rather than human studies. This scoping review assessed literature on perinatal e-cigarette use by pregnant mothers and its physiological and morphological consequences for children aged 0-3 years by systematically reviewing peer-reviewed publications from January 2013 to December 2023 using EMBASE, Ovid MEDLINE, and Web of Science databases, ultimately including eight studies after applying the Joanna Briggs Institute tools from an initial 32 studies meeting inclusion criteria. Results demonstrated that nicotine crosses the placental barrier, allowing measurement of its primary metabolite, cotinine, in the fetus. Studies in fetal mice indicate that nicotine exposure during development may lead to alterations in jaw and facial morphology. The evidence indicates that perinatal e-cigarette exposure negatively impacts newborn craniofacial and pulmonary development and can result in stillbirth and life-threatening consequences in both human and animal subjects, suggesting future research should expand fetal measurements to include accurate levels of active nicotine metabolites like cotinine to provide physicians insight into substance thresholds that produce specific abnormalities.

## Introduction and background

E-cigarettes, commonly referred to as “vapes'' or “nic sticks,” are one of the most common forms of nicotine devices used to simulate the action of smoking [1]. The surge in use of e-cigarettes began in 2007 with the hope that this would aid in smoking cessation [2,3]. While there is evidence that vaping devices containing nicotine can increase quit rates [4], there has been a growing use of vaping devices in both minors and adults who have no history of traditional cigarette usage, as well as severe systemic complications due to them containing heavy metals and other harsh chemicals, such as acetone [2]. E-cigarettes have gained popularity due to factors such as cost, variety of flavors, accessibility, and the influence of social media [3], but have been increasingly associated with negative health repercussions in organs including the brain, heart, and lungs**.**

Though vaping devices can be constructed in a variety of different styles, typically they consist of a heating apparatus, an energy supply (typically a lithium battery), and a receptacle for the “vapor fluid” [5]. However, the appeal of vapor devices, especially to adolescents, is attributed to enticing flavors and naivety about the possible health outcomes. Recent data from 2015 showed that 99.04% of e-cigarettes sold at convenience stores, supermarkets, mass merchandisers, drug, club, and dollar stores, and Department of Defense commissaries in the USA contained nicotine, while only 0.36% contained zero nicotine and 0.59% had a nondiscernible nicotine content [6]. Additionally, recent studies have challenged the notion that vapor devices are entirely without health risks. Nicotine, while largely responsible for the addictive properties of e-cigarettes and vapes, is not solely responsible for what makes them a controversial device today. These vapes contain moisture-preserving substances, such as propylene glycol or vegetable glycerin, which have been shown to be carcinogenic when inhaled [7,8]. In addition, these chemicals undergo thermal degradation into aldehydes. These substances can alter physiological processes and produce airway irritation [9]. Investigations have shown a J-shaped association of benzaldehyde with cardiovascular disease (CVD), as well as a positive linear association between isopentanaldehyde and CVD [10]. Even with this knowledge, vaping remains highly prevalent.

The use of e-cigarettes has continued to increase throughout the years among teenagers who have never smoked and adults who previously smoked [11]. In a study conducted by Jamal and his colleagues, adults aged 18-24 had the greatest decline in cigarette smoking use; however, they were also found to have increased use of e-cigarettes [12]. Cigarette smoking among pregnant women is overall decreasing, but younger women ages 20-24, American Indian/Alaska Natives, and those with the least education (General Education Development or high school diploma) have the highest prevalence [13]. The prevalence of vaping during pregnancy in a survey of 4500 women who gave birth suggested that overall rates were around 2.8%. However, in populations of women who have previously smoked, the percentage was a disproportionately high 17.7%, reinforcing the point that the addictive substances in e-cigarettes can have significant impacts on prevalence [14]. Unfortunately, there are misconceptions about the safety of vaping devices during pregnancy, with expectant mothers proving to be unaware or uncertain about the presence of nicotine in e-cigarettes and thus believing that e-cigarettes pose less harm to the unborn child compared to conventional cigarettes [15]. In fact, some women report using e-cigarettes to decrease or stop smoking to improve their own and their baby's health [16]. Additionally, pregnant women are frequently exposed to both tobacco and e-cigarettes, typically through their partners [17,18].

In order to ethically investigate e-cigarettes through experimental research, animal models are increasingly utilized for investigating their potential effects on cardiovascular health, neural development, and subject behavior. Initial findings from animal models demonstrated that exposure of rat mothers to aerosols from e-cigarettes with and without nicotine resulted in abnormal vascular development in three- and seven-month-old offspring, with associated impaired aortic relaxation. However, the use of medications like Febuxostat, a xanthine oxidase enzyme inhibitor, can reverse this impairment, indicating the involvement of reactive oxygen species (ROS) [19]. In the lungs of mouse model offspring in utero, pulmonary development itself can be affected through exposure to the aerosol from mint-flavored JUUL™, a common brand of e-cigarette, causing drastically altered gene expression associated with allergy and asthma [20]. In a study using pre-gravid rats that were subjected to the contents of e-cigarette vapor, higher protein kinase activity altered brain activity through modified protein synthesis and autophagy. Higher incidence of these proteins within fetal hippocampi resulted in reduced fetal weight, highlighting the influence of vaping with e-cigarettes on brain activity and the downstream effects on metabolism [21]. In addition, offspring of female mouse models that were exposed to aerosols from e-cigarettes containing nicotine during pregnancy expressed higher levels of DNA methylation in 13 genes associated with modulating neurological activity. These offspring showed increased deficits in anxiety, short-term memory, and hyperactivity, suggesting that the use of e-cigarettes during pregnancy can lead to both epigenetic and cognitive changes in the progeny [22]. Furthermore, a murine model study examining neurotoxicity of e-cigarette vapor during early development found reductions in certain hippocampal gene expression as well as upregulation of markers indicative of proinflammatory microglia, emphasizing the risk that vaping device aerosols present to the maturing central nervous system [23]. Further analysis has revealed an increase in oxidative stress, fibrotic tissue formation, and inflammation in the development of renal tissue in offspring of maternal mice who received e-cigarette vapor containing nicotine [24]. Understanding the impact of nicotine on neuronal, cardiac, and pulmonary development in animal models holds significant promise for unraveling the complex consequences of nicotine exposure on multiple organ systems, providing essential insights for public health policies and therapeutic interventions. This study aims to compare findings from human and animal studies to better understand the impact of prenatal e-cigarette exposure on fetal and early childhood development.

## Review

Methods

Eligibility Criteria

To meet inclusion criteria, articles had to be published in English, peer reviewed, and conducted between January 1, 2013, and September 30, 2023. These dates were selected to ensure data were current and relevant to the modern era of e-cigarette products in the market. In addition, articles were required to include information about pregnancies in human mothers at least 18 years of age who used electronic cigarettes during their pregnancy or any animal model exposed to e-cigarettes or nicotine during pregnancy. Congenital effects on human offspring were evaluated in patients from 0 to 3 years of age. This age range was selected because our study specifically aimed to evaluate congenital defects that could have been produced due to maternal e-cigarette usage. Effects on animal offspring could be evaluated at any time from in utero until after birth. We chose to limit our studies to industrialized Western countries with similar access to tobacco products.

Search Strategy

The search strategy was modeled around the Population, Context, and Concept (PCC) strategy. Our population included pregnant patients who were at least 18 years of age or pregnant animal models of any age and their offspring that were exposed to e-cigarette use prenatally. Our concept addressed the physical health effects of e-cigarette use by adults on children aged 0-3 years or the offspring from an animal model, and our context included studies that were performed in industrialized Western countries.

The following defined search terms were used to capture both human studies and animal models: “pregnant women” AND “tobacco use” OR “electronic cigarette” AND “fetus” OR “developmental disorder” OR “pregnancy disorder” OR “congenital disorder” AND “postpartum”. All components of the search strategy (including index terms and keywords) were adapted to fit each database.

Databases utilized in the search included Ovid MEDLINE, EMBASE, CINAHL Complete, and Web of Science. All searches were performed on October 17, 2023.

Selection of Sources of Evidence

Citations were compiled and uploaded into EndNote 21.0.1 / 2023 (Clarivate Analytics, Philadelphia, PA, USA) to perform the deduplication process. Titles and abstracts were screened for assessment on whether the inclusion criteria were met. Sources deemed potentially relevant were further analyzed in full, and their citation details were imported into the JBI System for the Unified Management, Assessment and Review of Information (JBI SUMARI; www.sumari.jbi.global). Two reviewers assessed the full text of each of the citations in detail against the inclusion criteria. These reviewers recorded and reported any reasons for exclusion. Conflicts that arose between reviewers were resolved with discussion or with the opinion of a third reviewer. All reviewers were blinded during the screening process and unblinded to resolve any conflicts. The results of the search and inclusion process were reported in the form of a Preferred Reporting Items for Systematic Reviews and Meta-analyses extension for Scoping Review (PRISMA-ScR).

Data Charting Process

Data were extracted using a data extraction template developed by the authors. The data that was extracted included specific details about the population, concept, and context, as well as significant findings and methods utilized in the articles. The authors used Microsoft Excel (Microsoft Corporation, Redmond, WA, USA) to extract and organize data. A third reviewer was utilized to resolve inconsistencies.

Critical Appraisal

The methodological quality and risk of bias of the included studies were assessed using the Cochrane Risk of Bias tool (RoB 2), which evaluates potential bias across five domains: bias arising from the randomization process, bias due to deviations from intended interventions, bias due to missing outcome data, bias in measurement of the outcome, and bias in selection of the reported result. Each domain was rated as low risk of bias, some concerns, or high risk of bias, and an overall risk of bias judgment was assigned for each study based on these domain-level assessments. Two reviewers independently assessed each study, with disagreements resolved through discussion or consultation with a third reviewer.

Results

**Figure 1:**
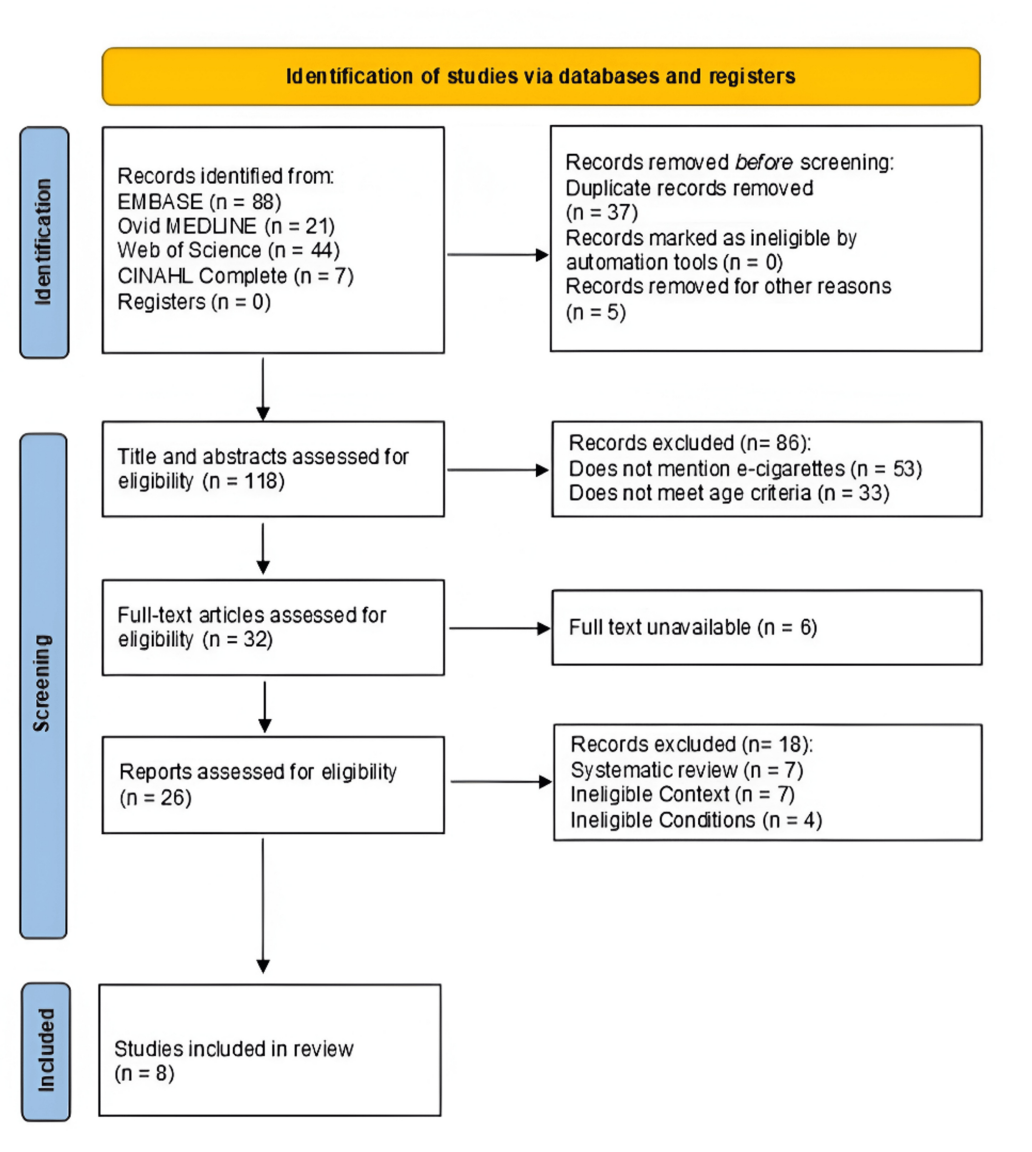
PRISMA-ScR flowchart detailing the article selection process PRISMA-ScR: Preferred Reporting Items for Systematic Reviews and Meta-analyses extension for Scoping Review

A search of the databases utilizing the predetermined search terms returned 160 articles. Eight articles remained after removing duplicates and applying the inclusion and exclusion criteria as detailed in the PRISMA-ScR chart (Figure 1). A critical appraisal was conducted on these articles with the determination of bias displayed in Table 1. While four of the eight articles indicate a high risk of bias, largely due to nonrandomized designs and reliance on self-reported exposure data, many studies maintained strong methodological rigor in other assessed domains. A summary of each article based on its purpose, study design, study population, methods, limitations, and key findings was tabulated in Table 2. The results from the studies indicate that prenatal exposure to nicotine, whether from e-cigarettes or traditional cigarettes, has significant effects on fetal development. Notably, nicotine exposure was associated with craniofacial abnormalities, impaired lung development, and increased risks of birth defects, fetal death, and low birth weight. Additionally, exposure to flavored e-cigarettes, particularly mint and menthol flavors, showed a higher risk of adverse fetal outcomes. The findings also suggest that nicotine may exacerbate the effects of other chemicals in e-liquid mixtures, although specific chemicals responsible for these outcomes were not fully identified. Limitations across studies, such as small sample sizes, lack of randomization, and absence of long-term follow-up, call for further research to better understand the full impact of prenatal nicotine exposure.

**Table 1:** Cochrane Risk of Bias Assessment of studies investigating the effects of e-cigarette exposure during pregnancy This table summarizes the methodological quality and potential biases of selected studies examining the outcomes associated with prenatal e-cigarette exposure. Risk of bias was assessed across multiple domains according to the Cochrane Risk of Bias Tool [33].

Study	Bias from the randomization process	Bias due to deviations from intended interventions	Bias due to missing outcome data	Bias in the measurement of the outcome	Bias in the selection of the reported result	Overall risk of bias	Justification
Froggatt et al. (2020) [25]	High	Some concerns	Some concerns	High	Some concerns	High	Non-randomized design with self-reported exposure data and a lack of socioeconomic status adjustment.
Kennedy et al. (2017) [26]	Some concerns	Low	Low	Low	Low	Some concerns	Quasi-experimental design without randomization; however, controlled laboratory conditions minimize other biases.
Lin et al. (2023) [27]	Some concerns	High	High	High	Some concerns	High	Retrospective cohort with self-reported data, missing information, and potential selection bias.
Ozekin et al. (2023) [28]	Some concerns	Low	Low	Low	Low	Some concerns	Quasi-experimental animal study with controlled exposure; however, the lack of randomization introduces some concerns.
Kishinchand et al. (2024) [29]	Some concerns	Some concerns	Low	Some concerns	Some concerns	Some concerns	Quasi-experimental design with variability in exposure and potential measurement biases.
Lavezzi et al. (2022) [30]	Some concerns	Low	Low	Low	Low	Some concerns	Quasi-experimental design with thorough postmortem analyses; however, the lack of randomization remains a concern.
Cohn et al. (2023) [31]	Some concerns	High	Some concerns	High	Some concerns	High	Longitudinal cohort with self-reported data and a small sample size for exclusive e-cigarette users.
Shelke et al. (2023) [32]	High	High	Some concerns	High	Some concerns	High	Cross-sectional design with self-reported data and limited generalizability due to the non-participation of some states.

**Table 2:** Data extraction chart This table summarizes the data extracted from all articles included in this review. CDC-PRAMS: Centers for Disease Control and Prevention-Pregnancy Risk Assessment Monitoring System; ENDS: electronic nicotine delivery system; NBAS: Neonatal Behavioral Assessment Scale; PATH: Population Assessment of Tobacco and Health; PRAMS: Pregnancy Risk Assessment Monitoring System; SIUDS: sudden intrauterine unexplained deaths

Authors	Purpose	Study design	Study population	Methods	Limitations	Key findings
Froggatt et al. (2020) [25]	To explore how prenatal e-cigarette exposure compares to prenatal cigarette exposure	Case-control study	83 infants, either exposed prenatally to cigarettes or e-cigarettes or not exposed to either	Birth outcomes and scores on the NBAS at one month of age were assessed and compared among the different infant groups	These results were collected on a self-report basis, so it is difficult to quantify how much nicotine was truly utilized. In addition, this study did not assess a person’s socioeconomic status and its potential effects.	When compared to non-exposed infants, cigarette-exposed infants had a significantly lower birth weight, while e-cigarette-exposed infants did not differ in birth weight, gestation, or head circumference.
Kennedy et al. (2017) [26]	To investigate whether chemicals produced by e-liquid aerosolization can adversely affect embryonic development, in particular craniofacial formation	Quasi-experimental study	Embryos from *Xenopus laevis* species (frog) collected from two different females	Embryos were collected and placed into two treatment groups differing in e-cig exposure time during development. All embryos were incubated throughout treatment until stage 43-45, where further analyses were conducted.	The use of *X. laevis* embryos limits generalizability to human development, and the collection of embryos from only two females reduces genetic diversity in the sample. Additionally, the quasi-experimental design lacks full randomization, which may introduce bias. The study also did not assess long-term outcomes beyond early development or conduct a detailed dose-response analysis, and the specific chemical components responsible for the observed craniofacial defects were not fully identified.	Aerosolized e-liquid mixtures (E-CigAM) exposure during the two developmental timelines had significant craniofacial defects, including eye abnormalities, midface hypoplasia, and median cleft. It was found that nicotine was not the main contributor to craniofacial defects, but it exacerbates the effects of the other e-liquid compounds.
Lin et al. (2023) [27]	To examine factors that may be associated with behavioral changes in e-cigarette use before and during pregnancy, to evaluate the association between e-cigarette use before and during pregnancy and adverse birth outcomes, and to explore the association between vape flavorings and adverse birth outcomes	Retrospective cohort study	23,301 women of reproductive age (18-44 years old) collected from a national sample of tobacco users and non-users via the PATH study	Data were collected from the PATH study. Based on this data, the researchers collected data on high-risk births and fetal defects (dependent variable) and compared them to various independent variables, such as social factors, personal beliefs, health status, and cigarette smoking status	The analytical sample size of pregnant women who vaped e-cigarettes was small. Some data were missing, which may indicate selection bias. Some women may have skipped questions for various reasons, leading to underreporting of certain variables.	More research is needed on behavioral choices. Opting to quit vaping before pregnancy showed no difference in the likelihood of having a high-risk birth. Women who smoked mint/menthol flavors showed a higher risk of experiencing fetal death than those who smoked other flavors.
Ozekin et al. (2023) [28]	To test the hypothesis that moderate daily exposure to nicotine vapor during the entirety of gestation disrupts fetal lung and skeletal development assessed at E18.5, the last day of in utero mouse development	Quasi-experimental study	Genetically altered mouse models, with and without nicotine, affected potassium channels (important in craniofacial and axial skeleton development)	The pregnant mice were exposed to nicotine vapor for four hours per day starting at 0.5 days until 18.5 days in utero. Mouse embryos were collected, and the skull, limbs, and lungs were assessed for any abnormalities.	The use of a mouse model may not fully replicate human fetal development. The quasi-experimental design without randomization could introduce bias. The study exposed the mice to a single nicotine vapor dosage and duration, limiting the understanding of dose-response effects. Additionally, there was no postnatal or long-term follow-up to assess potential effects after birth. The study also did not isolate specific chemicals in the nicotine vapor responsible for the observed developmental effects.	Maternally vaped embryos had histologic and transcriptional changes similar to those of impaired distal lung damage. Vaped embryos had reduced maxilla, palatal shelves, humerus, and femur lengths. KCNJ^KO/+ mice have significantly lower birth weights
Kishinchand et al. (2023) [29]	To establish a model for the study of ENDS components on craniofacial development. Overall efforts are needed to understand how the formulation of the common carrier components nicotine and PG/VG affects craniofacial development with these emerging technologies.	Quasi-experimental study	8-year-old sexually mature male and female mice	All female dams were exposed to nicotine using one puff/minute, four hours/day, five days/week throughout gestation. Litters were allowed to age until day 15 postnatally, then sacrificed to study cephalometric parameters and weight.	Not clearly identified. They make note that ENDS show markedly different chemistries, varying nicotine concentration amounts, and varying materials to allow for vaporization. Implies that the amount of nicotine exposure varies depending on the ENDS.	Suggests alterations to several facial morphology parameters in the developing offspring, then suggests that using ENDS during pregnancy could alter facial growth.
Lavezzi et al. (2022) [30]	To evaluate in each case the main neuronal vital centers of the brainstem and the lung developmental phase with respect to the gestational age and to correlate the results with the identified risk factors.	Quasi-experimental study	67 fresh antepartum stillbirths. Divided into (1) SIUDS with delayed pulmonary development for gestational week, a pathological finding not incompatible with prenatal life, (2) SIUDS with normal lung development for gestational age, and (3) stillbirths with cause of death determined by autopsy and used as controls	Information for each case was collected through questionnaires for risk factors associated with stillbirths. Then, a comprehensive fetal postmortem exam was conducted, including umbilical cord/placental/membrane evaluation and a careful analysis of the pulmonary developmental stage.	The sample size of 67 cases may not be fully representative of the broader population. The study relied on retrospective questionnaires, which could introduce recall bias. It mainly focused on tobacco smoke exposure, limiting the consideration of other potential risk factors. There was no long-term follow-up to assess post-birth outcomes. Randomization introduces potential bias.	Believed that there is a specific relationship between intrauterine tobacco smoke exposure and the delayed maturation of fetal lungs. Overall, this encompasses the development of lungs, respiratory activity in utero, and their control of specific nerve centers in sudden unexplained fetal deaths. Nicotine is suggested to be a major risk factor involved in the pathogenesis of pulmonary hypoplasia in stillbirths.
Cohn et al. (2023) [31]	To examine the prevalence and correlates of past 30-day exclusive e-cigarette use and dual-use of e-cigarettes with other tobacco products in a sample of currently pregnant women and investigate associations between past 30-day e-cigarette and other tobacco use with pregnancy and birth outcomes	Longitudinal cohort study	1037 women aged 18 years or older, who were currently pregnant during Waves 1 to 4 and had pregnancy and live birth outcome data in the subsequent wave	Each wave (1-5) in the published PATH studies was considered a baseline for the previous wave. Analyses compared associations between past 30-day tobacco use assessed during pregnancy with adverse pregnancy outcomes	The representative sample size was small, and it was difficult to examine exclusive e-cigarette use. Unable to access the frequency and quantity of e-cigarette or other tobacco use during pregnancy. Underreporting of tobacco use during pregnancy may be possible. Examining e-cigarette use over a 30-day span might capture very light usage and may underestimate the risks.	Compared to non-tobacco use, past 30-day tobacco use during pregnancy was not associated with increased odds of an adverse pregnancy or birth outcome. Past 30-day NON-e-cigarette use was associated with increased odds of an adverse pregnancy outcome, but not an adverse live birth outcome.
Shelke et al. (2023) [32]	To assess both the individual and interactive effects of prenatal depression, diabetes, and smoking in pregnant women on infant birth defects	Quasi-experimental study	89,839 women who had a recent live birth who responded to the PRAMS mail questionnaire or participated in the PRAMS phone survey	Data from the CDC-PRAMS for 2018 were used to make new subsets for analysis. The independent variables, depression, smoking, and diabetes, were identified, and then the dependent variable, birth defect, was identified as present or absent. The diabetes subset included all types. Data were analyzed and compared.	The cross-sectional design restricted the ability of temporality between the independent variables of smoking, diabetes, and depression during pregnancy on birth defects. The information was self-reported and may have led to information bias. A few states did not participate in the CDC-PRAMS 2018, which does not make the information applicable to all of the USA. Diabetes was listed as one category rather than being divided into type 1, type 2, and gestational. The article is not always clear whether they are discussing smoking traditional cigarettes alone or both traditional cigarettes and e-cigarettes as a single category.	Depression is strongly associated with having an infant with some form of birth defect. Depression also had a significant interaction with smoking and diabetes. One piece of advice from the authors was that reducing the number of pregnant women with depression may help lower birth defects among infants.

Populations

Five of the eight studies included human subjects [25,27,30-32]. The human subjects included the following: women who were of reproductive age and willing to share their background information and those who were currently pregnant or were pregnant at the time of the study [27,31,32], and infants with nicotine exposure during development [25,30]. The remaining three studies involved animal subjects, such as mice [28,29] and frog species [26]. The mice populations compared included one group that was sexually mature with protein channel mutations [28] and another that remained genetically intact [30]. Interestingly, the frog species utilized, *Xenopus laevis*, was advantageous in letting researchers better assess craniofacial development due to their large size, rapid development, and numerous progeny [26].

Methodology

The methodology of the articles generally followed a similar pattern. Notably, they each included a confirmation that the mother was previously pregnant or pregnant at the time of questioning; verification that subject inclusion criteria were met; and data on the mother’s nicotine exposure, along with her background information. From these guidelines, each study analyzed nicotine’s effect on the offspring [25,27,30-32].

Animal studies followed a typical experimental design in three of the identified papers [26,28,29]. This design involves manipulating one or more independent variables to observe their effects on dependent variables while controlling other factors to establish causal relationships. The pups of the mice [29,30] and embryos of *X. laevis* [26] were treated with nicotine pre- and post-gamete fertilization. After offspring birth or embryo development, the offspring were examined for developmental abnormalities in the skull [26,28,29].

Nicotine crossing the placenta: Two articles tested the effects of tobacco use and its impact on fetal development [25,30]. Nicotine crosses the placenta in humans; however, fetal nicotine levels can be up to 15% higher than those in the mother [28]. Nicotine has an optimal hematic pH of 7.4, in addition to being a fat-soluble substance, allowing nicotine to cross the placenta and fetal blood-brain barrier [30]. Furthermore, pregnant mice exposed to e-cigarette vapor containing 2.4% nicotine using a Tegue-TE2 smoking machine caused the mother’s plasma cotinine levels to increase to 35.12 ± 6.59 ng/mL [28].

Increased risk of adverse fetal outcomes: Three articles examined data from large-scale data systems [27,31,32]. The Population Assessment of Tobacco and Health (PATH) study is a widespread study that collects data from US tobacco and non-tobacco users and allows for studies to compare the effects of tobacco on various personal characteristics [27,31]. The CDC-Pregnancy Risk Assessment Monitoring System (CDC-PRAMS) was a questionnaire or phone survey given to women who had a recent live birth and included prenatal care, physical abuse, contraception, economic status, and early infant development [32]. These nationwide data systems allow researchers to collect data from a vast number of human subjects and compare select variables, such as social factors, health status, and nicotine exposure, to fetal defects. In two studies, e-cigarette usage was not associated with increased odds of adverse birth outcomes [27,31]. However, a significant association between smoking e-cigarettes was found with the CDC-PRAMS study [32], and use of mint/menthol-flavored e-cigarettes was associated with a significantly higher risk of fetal death in the PATH study [27]. Additionally, non-e-cigarette tobacco use, such as traditional cigarettes, was linked to a twofold increase in the incidence of adverse birth outcomes [31] as well as an increased risk of birth defects, though in the latter study the authors do not always make it clear if they are discussing traditional cigarette use alone or in a single category with e-cigarette use [32]. When considering social factors, it was found that interactions between smoking and prenatal depression or diabetes and prenatal depression displayed high odds of associated birth defects.

Two of the articles examined infants directly [25,30]. A case study assessed the neurobehavioral outcomes from nicotine exposure by testing infants using the Neonatal Behavioral Assessment Scale, concluding that both cigarette and e-cigarette exposure led to a significantly higher number of abnormal reflexes, worse self-regulation, and motor maturity [25]. However, when assessing birth outcomes, cigarette-exposed infants showed significantly lower birthweight and reduced head circumference in comparison to both the e-cigarette-exposed infants and the non-exposed infants. In contrast, an experimental study examined antepartum stillbirths and how their death correlated with identified risk factors, such as nicotine exposure, eventually concluding that there is a specific relationship between intrauterine tobacco exposure and pulmonary hypoplasia [30].

Craniofacial malformation: Craniofacial malformations have been attributed to nicotine exposure during fetal development in four studies [27-30]. In two studies, it was found that mouse fetuses exposed to nicotine delivered by electronic cigarettes had shorter facial lengths compared to the control [28,29]. Specifically, there was a significant reduction in both mandible length and mandibular ramus height in nicotine-vaping Kcnj2KO/+. Furthermore, there was also a decrease in palatal shelves within the same fetuses [28].

In addition, *X. laevis *embryos that were exposed to lab-grade e-cigAM (aerosolized e-liquid mixture) with 24 mg/mL of nicotine demonstrated positional changes in the mouth and ventral landmarks [26]. This prompted the study to perform morphometric and mouth shape analyses, which revealed orofacial shape changes but no changes in size. It was found that the distance between the eyes was reduced, but facial height was not significantly reduced [26]. In the same study, the *X. laevis *embryos were exposed to different e-cigarette liquids containing different flavors denoted as e-cigAM A-F. E-cigAM E and F had the most significant changes in orofacial size and shape compared to the fetuses exposed to e-cigAM A-D [27]. Exposure to E-cigAM E led to mouths that were 20% smaller in size and 70% more round [26]. Additionally, embryos exposed to E-cigAM F exhibited a 40% reduction in intercanthal distance and a 9% increase in facial height [26]. These results are consistent with their original findings of craniofacial malformations [28,29].

A final study measured the head circumference in human infants exposed to cigarettes and e-cigarettes. Infants exposed to cigarettes had significantly reduced head circumference, whereas, interestingly, infants exposed to e-cigarettes had no significant reduction in head circumference compared to both non-exposed and cigarette-exposed groups [25].

Discussion

Eight articles in this review, representing both human and animal studies, describe the implications of perinatal e-cigarette use by pregnant mothers in the United States on their offspring. Taken together, the studies indicated that exposure to nicotine and its metabolic subtypes may yield significant alterations in fetal morphology in both animal and human subjects. These effects can be stratified into findings regarding abnormal placental and craniofacial alterations.

Transplacental Transfer of Nicotine Causing Abnormal Placental Alterations

Most studies that have been conducted regarding nicotine crossing the placenta have evaluated tobacco products, which contain other teratogenic products [34]; however, studies that successfully evaluated nicotinic transmission through the placenta utilize cotinine, the primary metabolite of nicotine, as a measurement to quantify the transmission [28]. This exposure may be sufficient to disrupt placental development, impair vascularization, and alter gene expression, providing a mechanistic explanation for the observed associations between prenatal nicotine exposure and adverse outcomes such as fetal growth restriction, preterm birth, and congenital anomalies, including craniofacial defects [35,36]. Studies were also able to correlate nicotine as a significant prognosticating factor in the pathogenesis of pulmonary hypoplasia in stillbirths, suggesting that cross-placental transmission is evident [30].

Craniofacial Alterations

The development of craniofacial modifications is another significant finding associated with e-cigarette use. A recent systematic review and meta-analysis found a moderate association between maternal smoking and cleft lip and/or palate [37]. Furthermore, dysregulation of the MAPK signaling pathway and oxidative stress in tobacco smoke-induced craniofacial deformities has been demonstrated in both zebrafish and human embryonic palatal mesenchymal cell models [27]. Studies utilizing mouse fetuses exposed to nicotine via e-cigarettes reported significant reductions in both mandibular length and ramus height [28,29]. These studies also indicated a decline in palatal shelves in mouse fetuses subjected to nicotine exposure, further reinforcing the compound’s detrimental effects on craniofacial development [28]. Additional findings included changes in orofacial shape and a reduction in the distance between the eyes when mouse embryos were exposed to e-cigarette nicotine [26]. These data suggest that certain e-cigarette liquids impair craniofacial development [26]. In contrast, assessment of head circumference among human infants exposed to traditional cigarettes versus e-cigarettes showed no statistically significant difference between the two groups, suggesting possible global impairment of infant head development; however, future studies with greater power are needed to make these distinctions [25].

Limitations of Studies in the Review

One major limitation involved the measurements of cotinine, a metabolite of nicotine. A majority of the studies did not measure fetal plasma cotinine levels, which could have definitively shown that nicotine is able to pass through the placenta. The perceived negative social implications of nicotine use, especially during pregnancy, may serve as a deterrent to accurately reporting nicotine use, which may have limited the data in our selected articles.

This scoping review has a series of limitations that must be addressed in future studies. The variation in sample sizes and techniques in which the data were collected in the included studies may introduce inconsistencies in our findings. Moreover, this scoping review depends primarily on peer-reviewed articles published in English, which may have overlooked relevant studies in other languages or unpublished data. This limitation could result in a skewed representation of the existing evidence, favoring studies with statistically significant findings over null results.

The eligibility criteria for this scoping review made it necessary for the target population to be pregnant women aged 18 years or older who reported usage of e-cigarettes during the term of their pregnancy, as well as measuring effects among their offspring between the ages of 0 and 3 years old. This rigid inclusion framework leaves out investigations analyzing similar effects in larger populations like adolescents or non-pregnant adults, which might have given more perspectives into e-cigarette consumption trends and their concomitant impacts on maternal and child health alike.

Furthermore, the included studies vary in their methods, extending from observational cohort to quasi-experimental. This heterogeneity can introduce issues when trying to discern between the direct findings and may introduce inconsistencies in data interpretation. Alternatively, while epidemiological data systems like PATH and CDC-PRAMS are useful in some aspects of data collection, they lack minute data points on individual exposure levels or specific e-cigarette formulations. Studies such as the CDC-PRAMS study would benefit from a more careful classification of smoking traditional cigarettes versus smoking e-cigarettes, as the terminology used does not always make it entirely clear which they are addressing.

Finally, the review concentrated on nicotine exposure through e-cigarettes, but it did not thoroughly assess other factors such as socioeconomic status, environmental exposures, or co-use of other substances (i.e., alcohol or traditional cigarettes) that could affect maternal and child health outcomes. These additional elements might offer a more comprehensive interpretation of the noted health effects.

Future Directions

Future studies should begin by standardizing patient demographics, including but not limited to increasing sample size, diversifying enrollment across all races and ages, and juxtaposing findings with different modalities of nicotine acquisition besides e-cigarettes themselves. Studies may also be directed toward measuring levels of nicotine and its metabolites concurrently between the mother and fetus and determining if there is a true association or clinical threshold for negative effects on morphology. Performing these actions will provide a direct comparison of the placental, respiratory, and craniofacial effects as a result of perinatal nicotine exposure and assess the true impact of the compound on fetal morphology.

Despite the recognized limitations above, investigating this topic can yield tremendous clinical benefits in understanding the effects of nicotine on both the pregnant mother and the fetus itself. With effective communication, these results, coupled with traditional algorithms of management regarding smoking cessation, could result in reduced maternal intake of nicotine regardless of the delivery system. The findings can also be broadened to further assess the impact of perinatal e-cigarette usage across various nations outside of the United States to address the sweeping prevalence of tobacco usage across the world.

Subsequent research could additionally involve the implementation of longitudinal studies to observe the effects of perinatal e-cigarette usage in the newborn throughout their lifetime, across various ages and races, and measure any morphological changes that manifest in the subject. This approach will serve to provide a greater sense of understanding of the topic at hand under a lifespan approach.

## Conclusions

Perinatal e-cigarette exposure can negatively impact newborn craniofacial development as well as pulmonary development, which can result in stillbirth in both human and animal subjects. The increasing prevalence of e-cigarette usage, particularly in young adults of childbearing age, speaks to these findings having increasingly more relevant implications that translate to both preconception counseling and newborn medical care. Future studies should focus on expanding measurements to include accurate levels of active metabolites of nicotine, such as cotinine, in the fetus, which may provide insight into how much nicotine is required to cause each perinatal complication.
